# Estradiol upregulates voltage-gated sodium channel 1.7 in trigeminal ganglion contributing to hyperalgesia of inflamed TMJ

**DOI:** 10.1371/journal.pone.0178589

**Published:** 2017-06-05

**Authors:** Rui-Yun Bi, Zhen Meng, Peng Zhang, Xue-Dong Wang, Yun Ding, Ye-Hua Gan

**Affiliations:** 1 The Third Dental Center, Peking University School and Hospital of Stomatology, Haidian District, Beijing, China; 2 Central laboratory, Peking University School and Hospital of Stomatology, Haidian District, Beijing, China; 3 Center for Temporomandibular Disorders & Orofacial Pain, Peking University School and Hospital of Stomatology, Haidian District, Beijing, China; 4 The Department of Orthodontics, Peking University School and Hospital of Stomatology, Haidian District, Beijing, China; University of Kansas Medical Center, UNITED STATES

## Abstract

**Background:**

Temporomandibular disorders (TMDs) have the highest prevalence in women of reproductive age. The role of estrogen in TMDs and especially in TMDs related pain is not fully elucidated. Voltage-gated sodium channel 1.7 (Nav1.7) plays a prominent role in pain perception and Nav1.7 in trigeminal ganglion (TG) is involved in the hyperalgesia of inflamed Temporomandibular joint (TMJ). Whether estrogen could upregulate trigeminal ganglionic Nav1.7 expression to enhance hyperalgesia of inflamed TMJ remains to be explored.

**Methods:**

Estrous cycle and plasma levels of 17β-estradiol in female rats were evaluated with vaginal smear and enzyme linked immunosorbent assay, respectively. Female rats were ovariectomized and treated with 17β-estradiol at 0 μg, 20 μg and 80 μg, respectively, for 10 days. TMJ inflammation was induced using complete Freund’s adjuvant. Head withdrawal thresholds and food intake were measured to evaluate the TMJ nociceptive responses. The expression of Nav1.7 in TG was examined using real-time PCR and western blot. The activity of Nav1.7 promoter was examined using luciferase reporter assay. The locations of estrogen receptors (ERα and ERβ), the G protein coupled estrogen receptor (GPR30), and Nav1.7 in TG were examined using immunohistofluorescence.

**Results:**

Upregulation of Nav1.7 in TG and decrease in head withdrawal threshold were observed with the highest plasma 17β-estradiol in the proestrus of female rats. Ovariectomized rats treated with 80 μg 17β-estradiol showed upregulation of Nav1.7 in TG and decrease in head withdrawal threshold as compared with that of the control or ovariectomized rats treated with 0 μg or 20 μg. Moreover, 17β-estradiol dose-dependently potentiated TMJ inflammation-induced upregulation of Nav1.7 in TG and also enhanced TMJ inflammation-induced decrease of head withdrawal threshold in ovariectomized rats. In addition, the estrogen receptor antagonist, ICI 182,780, partially blocked the 17β-estradiol effect on Nav1.7 expression and head withdrawal threshold in ovariectomized rats. ERα and ERβ, but not GPR30, were mostly co-localized with Nav1.7 in neurons in TG. In the nerve growth factor-induced and ERα-transfected PC12 cells, 17β-estradiol dose-dependently enhanced Nav1.7 promoter activity, whereas mutations of the estrogen response element at -1269/-1282 and -1214/-1227 in the promoter completely abolished its effect on the promoter activity.

**Conclusion:**

Estradiol could upregulate trigeminal ganglionic Nav1.7 expression to contribute to hyperalgesia of inflamed TMJ.

## Introduction

Pain in the temporomandibular joint (TMJ) or masticatory muscles or both is one of the chief complaints of patients with temporomandibular disorders (TMDs). TMJ inflammation or synovitis is frequently observed in TMD patients and is the major reason for TMD pain [1, 2]. TMD has the highest prevalence in women of reproductive age, with a female-to-male ratio of more than 2:1, implying that estrogen may play a role in pain processing of TMD [3, 4]. Furthermore, we previously demonstrated that 17β-estradiol can aggravate TMJ inflammation through the NF-κB pathway [5] or enhance hyperalgesia of inflamed TMJ through upregulation of hippocampal TRPV1 in ovariectomized rats [6]. It appears that the mechanisms underlying estrogen involved in TMD pain would be complicated and remain to be fully understood.

Estrogens exert their effects through at least two different cellular mechanisms. One is the classical (genomic) mechanism of estrogen receptors (ER) action, which involves binding of estradiol to ERα or ERβ in the nucleus, after which the receptors dimerize and bind to estrogen response elements (ERE) located in the promoters of target genes and modulate their expression levels [7]. This “classic” pathway alters neuronal physiology and behaviour from hours to days after the initiation of hormonal manipulation [8]. The other is the “non-classical” or “non-nuclear” mechanism of estrogen action, which can activate rapid cytoplasmic signalling mediated by membrane-associated estrogen receptors (mER) [9] or membrane estrogen-binding sites such as GPR30 [10]. It can modify the neuronal cell excitability within seconds to minutes [8]. However, the mER and membrane estrogen-binding sites are not fully identified so far.

Voltage-gated sodium channel 1.7 (Nav1.7), encoded by a sodium channel voltage-gated type IX alpha subunit gene (SCN9A), is highly expressed in the dorsal root ganglia (DRG), trigeminal ganglia (TG), sympathetic ganglia [11]. Nav1.7 can amplify weak stimuli in the neurons and act as a threshold channel for firing action potentials [12]. Nav1.7 serves a remarkable function in pain perception. Mutations of this gene contribute to three human pain syndromes: primary erythromelalgia [13], paroxysmal extreme pain disorder [14] and congenital inability to experience pain [15]. Nav1.7 also plays an important role in inflammatory pain. Nav1.7 mRNA and protein in DRG are upregulated after carrageenan-induced inflammation in the hind paws [16]. An immunochemical study of sensory neurons in guinea pigs shows that Nav1.7 is associated with nociceptors [17]. Furthermore, knockdown or nociceptor-specific knockout of Nav1.7 can prevent inflammatory hyperalgesia [18, 19]. Our previous study also showed that mRNA and protein expressions of trigeminal ganglionic Nav1.7 were upregulated by TMJ inflammation, whereas trigeminal ganglionic Nav1.3 was not changed and trigeminal ganglionic Nav1.8 and Nav1.9 were only slightly upregulated [20]. Based on the importance of Nav1.7 in pain perception and inflammatory pain, we hypothesized that trigeminal ganglionic Nav1.7 might be involved in estradiol-enhanced TMJ inflammatory pain as described previously in detail [21].

In this study, we explored whether trigeminal ganglionic Nav1.7 expression was affected by estradiol, and furthermore whether estradiol could potentiate Nav1.7 expression in TG to contribute to hyperalgesia of inflamed TMJ.

## Methods

### Animals

Sprague–Dawley female rats (180–200 g, Vital River Laboratory Animal Technology, Beijing, China) were used in this study. The rats were allowed to acclimate for 7 days prior to experiments. The animals were kept in an air-conditioned room at a relative humidity of 40–60% and at a temperature of 22°C ± 1°C. The rats had free access to the standard laboratory food and sterilized water and were housed under a 12-h light/dark cycle. The rat room was kept clean, quiet, and uncluttered. Animals were monitored at least once daily, including weekends and holidays, and animals were monitored at least twice daily after recovering from anesthesia, or surgery for ovariectomy, or induction of TMJ inflammation, or replacement of estrogen, or estrogen receptor antagonist administration. In this study, four animals died under anesthesia based on the same anesthesia treatment (1% sodium pentobarbital, 40 mg/kg, i.p.) and the same care. However, it was possible that some of them may have died of individual sensitive responding to the anesthesia. No animals became severely ill. Animal housing, handling, experimental procedures, anesthesia and euthanasia conformed to the Guideline for Animal Care and Use Committee and Animal Experimentation of Peking University, and the experimental protocols were approved by the Peking University Biomedical Ethics Committee Experimental Animal Welfare Ethics Branch (NO.LA2008-004, Beijing, China). The food for rats contained only limited residual of soybean after oil extracted, its phytoestrogens might be neglected.

### Determination of estrous cycle

To evaluate whether TMJ nociception fluctuated during the estrous cycle of female rats, the estrus, metestrus, diestrus, and proestrus were determined with vaginal smear as described previously [22]. The vaginal smear samples were collected using the sterile cotton swab technique at 07:00–09:00 and 19:00–21:00 every day. The cotton-tipped end of this swab, which was moistened with sterile saline (0.9%), was passed into the vagina, and the swab was then rotated through a complete revolution in each direction and withdrawn. The cotton tip was rolled from one end of a glass microscope slide to the other end. The prepared smears were air-dried and then fixed at 4% paraformaldehyde for 10 min. The vaginal smears were stained with papanicolaou stain.

The stages of the estrous cycle were determined according to the cell types observed in the vaginal smear by a light microscope. A proestrus smear consists of a predominance of nucleated epithelial cells; an estrous smear primarily consists of anucleated cornified cells and often stacked together like a leaf; a metestrus smear consists of massive leukocytes and cornified epithelial cells, and a diestrus smear primarily consists of a predominance of leukocytes [22, 23]. Finally rats were determined for each phase of estrous cycle for behaviour test and blood collection.

### Animal group dividing and treatments

To evaluate the effect of estradiol on TMJ nociception without TMJ inflammation, female rats were randomly divided into 4 groups (n = 7): control group and ovariectomized rats treated daily with 17β-estradiol at doses of 0 μg, 20 μg, and 80 μg, respectively, for ten days. The control group was consisted of random cycling female rats. The 0 μg group received injection of vehicle instead of estradiol. To evaluate the effects of both estradiol and inflammation on TMJ nociception, female rats were randomly divided into 5 groups (n = 7): control group, sham-ovariectomized group, and ovariectomized rats treated daily with 17β-estradiol at doses of 0 μg, 20 μg, and 80 μg, respectively, for ten days. The last four groups were induced TMJ inflammation by complete Freund adjuvant (CFA) for 24 h. The control group and the sham-ovariectomized group were consisted of random cycling female rats. The control group received injection of vehicle instead of CFA, whereas the 0 μg groups received injection of vehicle instead of estradiol. To evaluate the effect of estrogen receptor inhibitor on TMJ nociception, female rats were randomly divided into 3 groups (n = 7): control group, TMJ inflammation group, and TMJ inflammation/ICI 182,780 group. The control group received injections of vehicles instead of CFA and ICI 182,780, whereas TMJ inflammation group received injection of vehicle instead of ICI 182,780.

### Behavioral testing

Behavioral testing was conducted on a blind basis, in which the investigators were blinded to the rat treatment. The head withdrawal threshold and food intake, which were negatively associated with the mechanical sensitivity of the orofacial region [24], were measured as previously described [6, 20, 25]. The head withdrawal threshold was calculated as mean ± standard deviation (SD) based on five measurements per joint and four rats per group. Briefly, the rats were habituated to rear on their hind paws and recline against the experimenter’s working glove. The rats can move freely, but were kept motionless during the test session. At 2 h before rats were sacrificed, the tip of the filament of electronic von Frey anesthesiometer (IITC Life Science, Woodland Hills, CA, USA) was placed on the skin above the TMJ with progressive, increasing forces to the TMJ region until the head was withdrawn; the applied force was automatically recorded.

### Food intake

Food intake was measured 4 hours before (baseline) and 20 hours after induction of TMJ inflammation as described previously [6, 20]. Briefly, each rat was isolated with supplying only water for 15 h. Then the food which has been weighed before was given to the rat for 2 h without supplying water to avoid wetting the food. The amount of uneaten food was weighed, and the amount of eaten food was calculated. Food intake was calculated as mean ± SD based on four rats per group.

### Estradiol administration

After being anesthetized with 1% pentobarbital sodium administered intraperitoneally, rats were bilaterally ovariectomized or sham-operated (control and sham ovariectomized groups) and allowed to recover for one week. The 17β-estradiol (Sigma) was dissolved in ethanol and diluted to 10% in saline immediately before administration. The ovariectomized rats were treated with 17β-estradiol by subcutaneous abdominal injection daily in the morning, at doses of 20 μg and 80 μg per rat, respectively, at a volume of 200 ml for 10 days. The control group, the sham-ovariectomized groups and the group with 0 μg of 17β-estradiol received subcutaneous abdominal injections of the same amount of vehicle [5, 6].

### Plasma hormonal determination

Blood was obtained from seven rats per group immediately after the experiments. The plasma levels of 17β-estradiol were measured by enzyme linked immunosorbent assay using an Estradiol EIA Kit (Cayman Chemical) according to the manufacturer’s instructions.

### Induction of TMJ inflammation

TMJ inflammation was induced as described previously [5, 20, 25]. Briefly, 50 μl of CFA (oil/saline at ratio of 1:1, 0.025 mg Mycobacterium tuberculosis, Sigma) was injected into bilateral TMJs to induce TMJ inflammation, and the control rats were injected with vehicle (50 μl saline) into bilateral TMJs.

### Application of estradiol receptor antagonist

The estradiol receptor specific antagonist, ICI 182780, was dissolved in 10% ethanol and intraperitoneally injected (500 μg in 100 ml) 24 hours before and immediately before induction of TMJ inflammation. The application and dose of ICI 182780 were used as our previous study [5].

### Real-time quantitative PCR

The bilateral TGs from four rats per group were dissected and pooled for total RNA isolation using TRIzol reagent (Invitrogen, Carlsbad, CA, USA). Reverse transcription were performed with an iScript cDNA synthesis kit (Bio-Rad) and real-time PCR was performed with Power SYBR Green PCR Master Mix (Applied Biosystems) using a 7500 real-time PCR System (Applied Biosystems) as previously described [20, 25]. The efficiency of primers for rat β-actin and rat Nav1.7 was confirmed previously [20], and the sequence was as follows: for rat β-actin, sense, 5’-TGA CAG GAT GCA GAA GGA GA-3’, antisense 5’-TAG AGC CAC CAA TCC ACA CA-3’; for rat Nav1.7, sense 5’-TCG TAC CCC ATA GAC CCC G -3’, antisense 5’-CTG ATT AGT CGT GCC GCT G -3’. All primers were custom-synthesized (Sangon Biotech Company, Shanghai, China).

### Western blot analysis

The bilateral TGs from three rats per group were dissected and pooled for homogenizing by a homogenizer (Ultra-Turrax T10, IKA Laboratory Technology) in an ice-cold denaturing lysis buffer (50 mM Tris-HCl, pH 7.5, 150 mM NaCl, 5 mM EDTA, 1% Triton X-100, 1 mM DTT, 1 mM phenylmethylsulfonyl fluoride, 1 μg/ml aprotinin, 1 μg/ml leupeptin) and centrifuged at 12,000 g for 30 min at 4°C. The supernatant was collected, and protein concentrations were determined via BCA assay (Pierce). Protein samples were subjected to 6–10% gradient sodium dodecyl sulfate–polyacrylamide gel electrophoresis (SDS-PAGE) and transferred to the PVDF membrane (Millipore, Bedford, MA, USA). The membrane was blocked with 5% bovine serum albumin (BSA, sigma, USA) in TBS-T buffer (50 mmol/L Tris–HCl, pH 7.5, 150 mmol/L NaCl, 0.05% Tween 20) for 1 h and incubated with anti-Nav1.7 antibody (1:500, AB5390, Millipore, USA) and anti-β-actin antibody (1:1000, sc-1616-R, Santa Cruz, USA) overnight at 4°C. After washing extensively with TBS-T, the membranes were incubated with horseradish peroxidase-conjugated secondary antibodies for 1 h at room temperature. The membrane was visualized using the ECL kit (NC14109, Thermo, USA) and Fusion system for Western blot and gel imaging (Fx, Vilber Lourmat, Marne-la-Vallée, France). The density of the immunoreactive bands was quantitated using the NIH Image 1.38 software. Nav1.7 expression was expressed as the fold change of the control group after normalization to β-actin.

### Immunohistofluorescence

Intact female rats were anesthetized with an overdose of pentobarbital sodium and euthanized by transcardiac perfusion (250 ml body temperature 0.1 M PBS pH 7.4, followed by 200 ml to 300 ml ice-cold 4% paraformaldehyde in 0.1 M PBS pH 7.4). After perfusion, TGs were postfixed in 4% paraformaldehyde for 4 h, incubated in 30% sucrose solution (in 0.1 M PBS) overnight at 4°C, frozen to -20°C, and sectioned 5 μm thick on a cryostat. The sections were then mounted on poly-L-lysine-coated slides, and used for immunohistofluorescence. Immunohistofluorescence was performed as described previously [5, 20]. For immunofluorescence, rabbit polyclonal anti-Nav1.7 antibody (1:500, AB5390, Millipore, USA), mouse monoclonal anti-Nav1.7 antibody (1:500, ab85015, abcam, UK), rabbit monoclonal anti-ERα antibody (1:200, ab32063, abcam, UK), rabbit polyclonal anti-ERβ antibody (1:2000, ab3577, abcam, UK)(its specificity has been well tested in previous study [26]), rabbit polyclonal anti-GPR30 antibody (1:200, ab39742, abcam, UK), mouse monoclonal anti-Neuronal Nuclei (NeuN, neuron marker) antibody (1:500, ab104224, abcam, UK), mouse anti-neurofilament200 (NF200, medium and large neuron marker) antibody (1:1000, ab82259, Abcam, UK), and mouse monoclonal anti-glial fibrillary acidic protein (GFAP, glia marker) antibody (1:300, P14136, Cell Signaling Technology, USA), were used correspondingly as primary antibodies. Double-immunostaining for ERα or ERβ or GPR30 with Nav1.7 was used to evaluate the co-localization of Nav1.7 and ERs. Double-immunostaining for NF200 with Nav1.7 was used to evaluate the location of Nav1.7 in different neurons as NF200 is the marker for medium and large neurons. Double-immunostaining for GFAP with ERα or ERβ was used to evaluate the location of ERα or ERβ in glia. Double-immunostaining for NeuN with ERα or ERβ was used to evaluate the location of ERα or ERβ in neuron as NeuN is the marker for neuronal nucleus. The cells in the same area (545Χ58 μm^2^) displaying immunoreactivity for ERα, ERβ, GPR30, or Nav1.7 were counted in three randomly selected fields on one section of each TG (total three TGs). Only the clearly labelled cells that presented a discernable nucleus or cytoplasm or both were included. The number of both ERα- and Nav1.7-positive neurons or both ERβ- and Nav1.7-positive neurons, and ERα-, or ERβ- or Nav1.7-positive neurons in each field was counted and the co-localized neurons was calculated as the percentage of the Nav1.7-positive neurons expressing ER or the percentage of ER-positive neurons expressing Nav1.7.

### Cells culture

PC12 cells contain ERβ and GPR30, but lack ERα [27, 28]; they are a suitable parent line for studying the effects of estrogen on neurons in vitro; they can be differentiated by NGF to better resemble a neuronal phenotype, and are easily transfected by mammalian expression vectors [29]. To examine the effect of estradiol on Nav1.7 promoter, PC12 cells were prepared with induction with NGF and transfection of ERα. PC12 cells were cultured in phenol red–free RPMI 1640 medium (GIBCO) supplemented with 5% (v/v) fetal bovine plasma and 10% (v/v) horse plasma at 37°C with 5% CO_2_. Twenty-four hours after the cells seeding in twelve-well plates coated by poly-lysine, the media were replaced and a recombinant NGF (50 ng/ml) was added for 48h for neuronal differentiation. Rat ERα plasmids (pEGFP-C1-ER alpha, ADDGEN) were transfected into the NGF-induced PC12 cell with Lipofectamine 2000 (Invitrogen) 24h before transfection of Nav1.7 promoter constructs.

### Report constructs

The sequence of the rat Nav1.7 promoter was obtained from GenBank. The putative promoter (-1489 bp to +49 bp) of rat Nav1.7 was amplified from the genomic DNA of rat liver with a high-fidelity DNA polymerase (TOYOBO) using standard PCR techniques. It was cloned into pGM-t vector (Invitrogen) and recloned into luciferase reporter plasmid at KpnI and XhoI sites using pGL3-enhancer vector (Promega). Confirmation was conducted by DNA sequencing. Primers with restriction enzyme sites (underlined) for cloning the Nav1.7 promoter were custom synthesized (Shanghai Sangon Biotech Co., Ltd, Shanghai, China): 5′-*GGTACCTGATTGACGCTTATTGG*-3′ (sense) and 5′-*CTCGAGTTGTGAAGTGAACGAAA*-3′ (antisense). The construct was confirmed by DNA sequencing. A series of mutants were constructed based on the promoter-reporter construct via PCR using DpnI enzyme. PCR was performed with a high-fidelity DNA polymerase (TOYOBO) as described previously [30]. The primers used for deleting the putative ERE (TGGCAgtaTGACC) at -452/-439 were as follows: 5′-CCATAGCAATAAAGTCATTGAGGTCATTGGGTGT-3′ (sense) and 5′-ACACCCAATGACCTCAATGACTTTATTGCTATGG-3′ (antisense). The primers used for deleting the putative ERE (GCTCAtctTGAAT) at -1227/-1214 were as follows: 5′-TTTGCAATATAGTCTATTCAAGATTTTTGCCTTA-3′ (sense) and 5′-TAAGGCAAAAATCTTGAATAGACTATATTGCAAA-3′ (antisense). The primers used for deleting the putative ERE (GGTCAagtTTGCT) at -1282/-1269 were as follows: 5′-TCCCTTAATACACAGGTGTTTGAATACTTTTTAA-3′ (sense) and 5′-TTAAAAAGTATTCAAACACCTGTGTATTAAGGGA-3′ (antisense). All primers were custom synthesized (Shanghai Sangon Biotech Co., Ltd., Shanghai, China). All mutant constructs were confirmed by DNA sequencing.

### Luciferase assay

The NGF-induced ERα-transfected PC12 cell were transfected with Nav1.7 promoter or the mutants using Lipofectamine 2000 (Invitrogen) for 4h when 17β-estradiol (10^−7^ moL/L) was added for another 24 h. Luciferase assay was performed as described previously [30]. Briefly, the transfected cells were lysed in a cell lysis buffer 28 h after the transfection. Luciferase activity was measured with a FB12 luminometer (Berthold, Germany) using luciferin as the substrate according to the manufacturer’s instructions (Promega).

### Statistical analysis

Statistical analysis was performed with SPSS 13 for Windows. All data were presented as mean ± standard deviation (SD). Measures for the head withdrawal threshold among groups were analyzed with repeated measures ANOVA. Differences between groups were examined by one-way ANOVA or two-way ANOVA. All multiple-group comparisons were followed by a Bonferroni post hoc test. Difference between two groups was examined using an independent samples *t* test. A value of *P* < 0.05 was considered statistically significant.

## Results

### Changes of Nav1.7 expression in TG and of TMJ nociception correlated with estradiol fluctuation in estrous cycle

**Fig 1:**
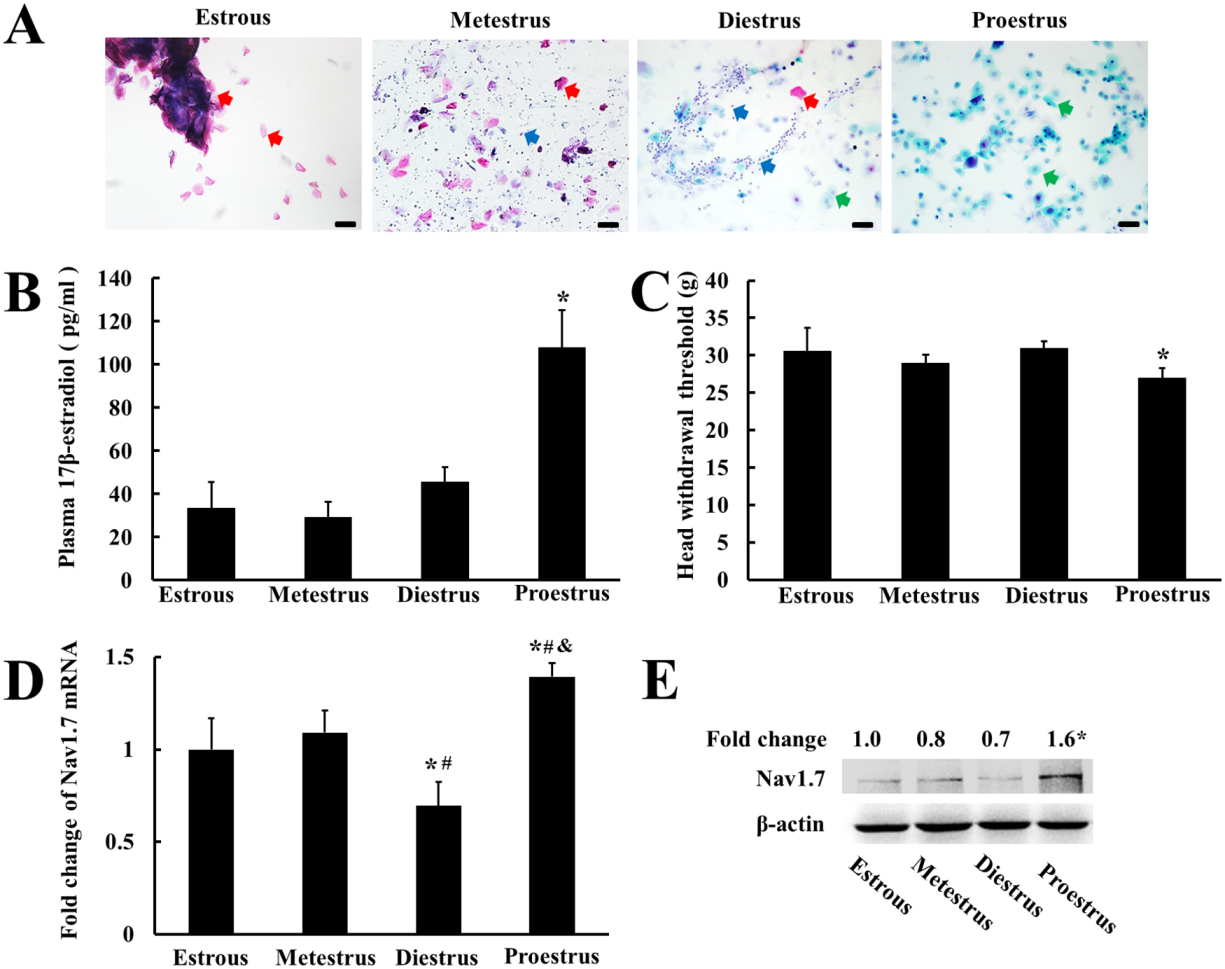
Changes in the expression of trigeminal ganglionic Nav1.7 and TMJ nociception were correlated with estradiol fluctuation in estrous cycle of female rats. (A) Photomicrograph of representative vaginal smears from intact cycling rats. Anucleated cornified cells (red arrow), nucleated epithelial cells (green arrow), leukocytes (blue arrow) were observed. Bars = 50 μm. (B) Plasma level of 17β-estradiol in female rats during estrus, metestrus, diestrus, and proestrus. The plasma level of 17β-estradiol was the highest in the female rat during proestrus. *P < 0.05 versus the rest groups. (C) Head withdrawal threshold during different estrous stages. The head withdrawal threshold was the lowest in the female rat during proestrus. *P < 0.05 versus the rest groups. (D) The mRNA expression of Nav1.7 in TG during different estrous stages. The mRNA expression of Nav1.7 was the highest in the female rat during proestrus.*P < 0.05 versus estrus group, ^#^*P* < 0.05 versus metestrus group, ^&^*P* < 0.05 versus diestrus group (n = 4, two-way ANOVA). (E) Representative immunoblotting for Nav1.7 expression in TG. The trigeminal ganglionic Nav1.7 protein expression corresponded to its mRNA expression pattern. β-Actin was served as an internal control for equal loading (n = 3). Data are expressed as mean ± SD.

To examine whether Nav1.7 expression in TG could be affected by physiological estradiol fluctuations, we measured head withdrawal threshold of TMJ and the mRNA and protein expressions of Nav1.7 in female rats during estrous cycles. The estrous cycles were determined by vaginal smears (Fig 1A). The plasma level of 17β-estradiol during estrus, metestrus, diestrus, and proestrus were 33.3 ± 12.3 pg/ml, 29.1 ± 7.1 pg/ml, 45.5 ± 6.8 pg/ml, and 107.8 ± 17.3 pg/ml, respectively, which was consistent with previous studies that the 17β-estradiol levels only significantly increased at proestrus [5, 31]. The plasma level of 17β-estradiol during the proestrus was highest (*P* < 0.05) among the estrous cycle (Fig 1B), whereas the head withdrawal threshold was correspondingly the lowest in the proestrus among the estrous cycles (*P* < 0.05) (Fig 1C). The mRNA and protein expressions of Nav1.7 were also correspondingly the highest in the proestrus and the lowest in the diestrus (*P* < 0.05) (Fig 1D and 1E).

### Estradiol dose-dependently upregulated Nav1.7 expression in TG and enhanced TMJ nociception in ovariectomized rats

**Fig 2:**
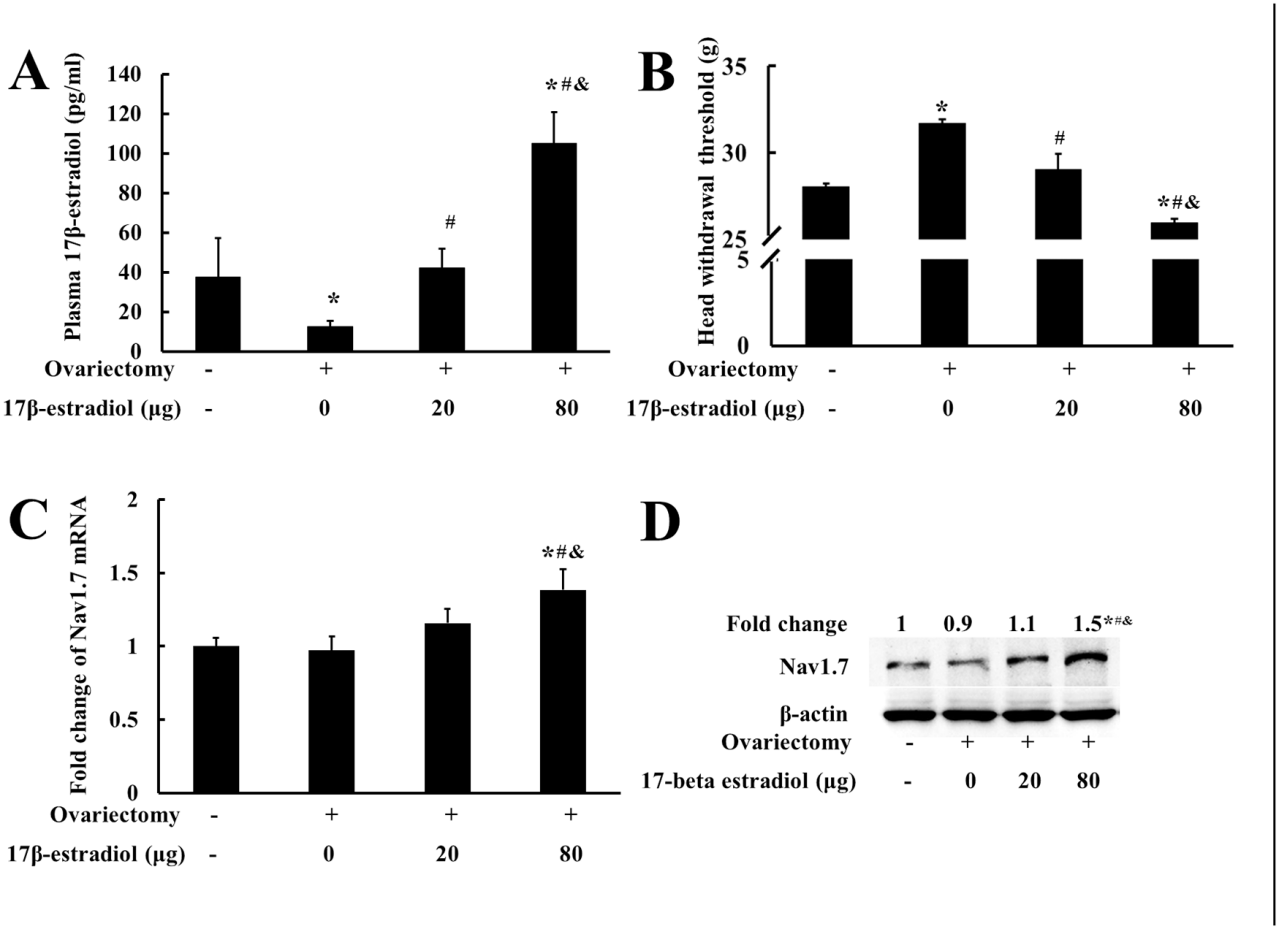
Estradiol dose-dependently upregulated Nav1.7 expression in TG and reduced the mechanical threshold in ovariectomized rats. (A) Plasma level of 17β-estradiol after estrogen replacement. (B) Head withdrawal threshold after estrogen replacement. (C) Nav1.7 mRNA expression in TG after estrogen replacement (n = 4, two-way ANOVA). (D) Representative immunoblotting for Nav1.7 expression in TG after estrogen replacement. β-Actin was served as an internal control for equal loading (n = 3). *P < 0.05 versus control group; ^#^*P* < 0.05 versus 0 μg group; ^&^*P* < 0.05 versus 20 μg group. Data are expressed as mean ± SD.

To further examine whether estradiol could upregulate Nav1.7 expression in TG and also enhanced TMJ nociception, female rats were divided into 4 groups: the control group (which received sham-operation of ovariectomy) and three ovariectomized group treated with 17β-estradiol at doses of 0 μg, 20 μg, and 80 μg, respectively, for 10 days as described previously [5, 6]. Body weight and plasma level of 17β-estradiol were measured to confirm the effectiveness of ovariectomy and 17β-estradiol replacement. The body weight of the ovariectomized group treated with 17β-estradiol decreased dose-dependently (data not shown), consistent with the previous study [32]. The plasma levels of 17β-estradiol was dose-dependently increased, and the plasma level of estradiol of the group replaced with 17β-estradiol at the dose of 0 μg was 12.7 ± 2.7 pg/ml, whereas the ovariectomized rats replaced with 17β-estradiol at the dose of 80 μg was the highest (105.8 ± 15.8 pg/ml), which was comparable to that of the proestrus of female rats (Fig 2A). In Fig 2B, the head withdrawal threshold was highest in the group treated with 0 μg of 17β-estradiol (*P* < 0.05) and was lowest in the group treated with 80 μg of 17β-estradiol (*P* < 0.05); the mRNA and protein expressions of Nav1.7 in TG was also highest in the group with 80 μg of 17β-estradiol (*P* < 0.05) (Fig 2C and 2D). The results suggested that upregulation of Nav1.7 expression in TG and increase in TMJ nociception might be related to plasma level of 17β-estradiol.

### Estradiol enhanced hyperalgesia of inflamed TMJ and further potentiated TMJ inflammation-induced Nav1.7 expression in TG in ovariectomized rats

**Fig 3:**
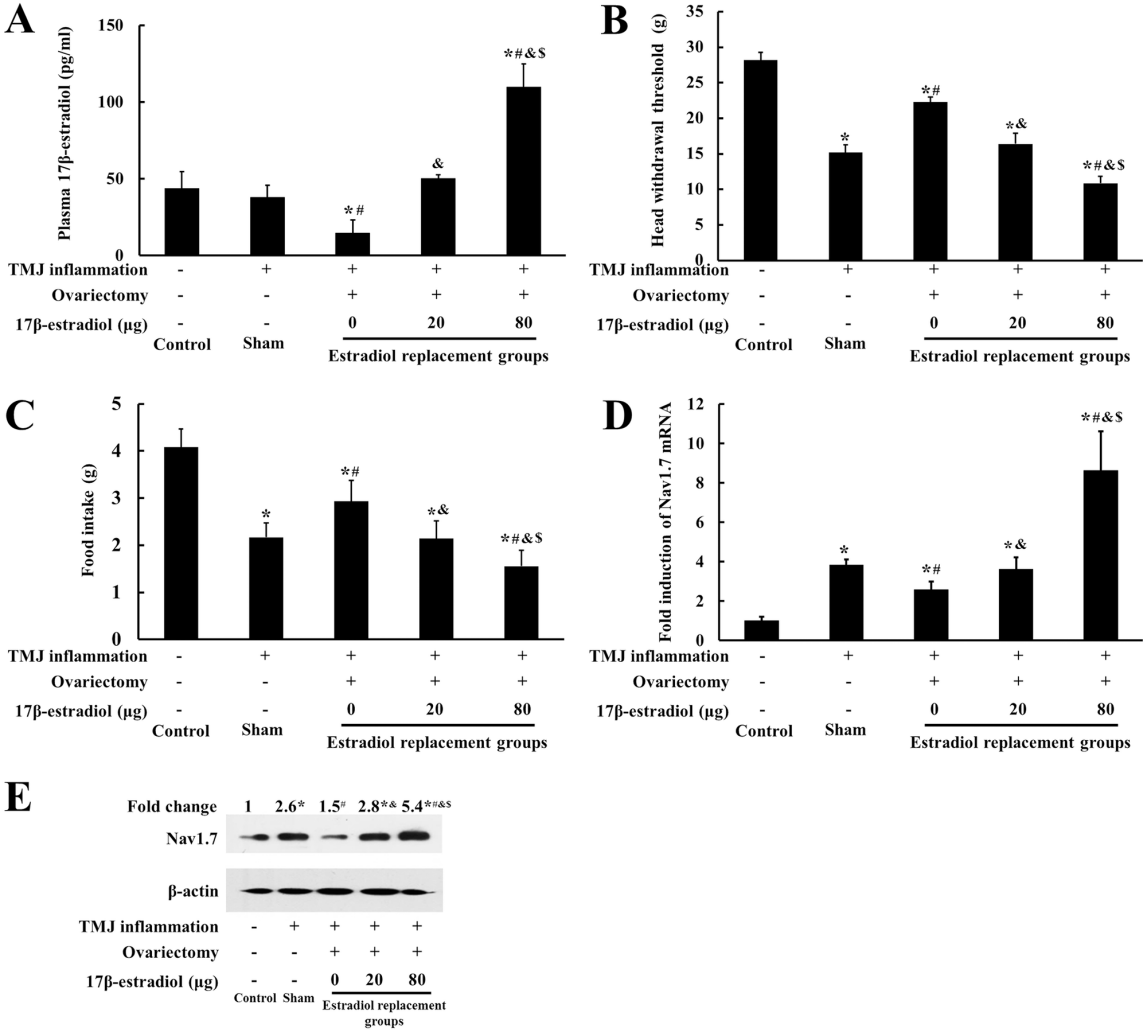
Estradiol dose-dependently reduced the mechanical threshold of inflamed TMJ and further potentiated TMJ inflammation-induced trigeminal ganglionic Nav1.7 expression. (A) Plasma level of 17β-estradiol. (B, C) The head withdrawal threshold and food-intake. (D) Nav1.7 mRNA expression in TG. The mRNA expression of Nav1.7 was upregulated by TMJ inflammation and further potentiated by 17β-estradiol in a dose-dependent manner (n = 4, two-way ANOVA). (E) Representative immunoblotting for Nav1.7 expression in TG. The trigeminal ganglionic Nav1.7 protein expression corresponded to its mRNA expression pattern. β-Actin was served as an internal control for equal loading (n = 3). *P < 0.05 versus control group; ^#^*P* < 0.05 versus sham group, ^&^*P* < 0.05 versus 0μg group; ^$^*P* < 0.05 versus 20μg group. Data are expressed as mean ± SD.

To examine whether trigeminal ganglionic Nav1.7 expression was correlated with estradiol-enhanced nociception of inflamed TMJ, rats were randomly divided into 5 groups: the control group, the sham-ovariectomized group, and 3 groups of ovariectomized rats treated with 0 μg, 20 μg, and 80 μg of 17β-estradiol, respectively, and the TMJs of the latter four groups were induced inflammation. Body weight (data not shown) and the plasma levels of 17β-estradiol were measured to confirm the effectiveness of ovariectomy and estradiol replacement. As shown in Fig 3A, the plasma levels of estradiol in the ovariectomized groups replaced with increasing doses of estradiol were increased dose-dependently.

As shown in Fig 3B, the head withdrawal threshold was decreased in all the CFA-treated groups compared with the control group (*P* < 0.05), and the head withdrawal threshold in the ovariectomized groups was decreased when the doses of 17β-estradiol increased (*P* < 0.05). Before TMJ inflammation was induced, food intake was no difference among all groups (data not shown). However, food intake was decreased in all the CFA-treated groups as compared with the control (*P* < 0.05), and food intake in the ovariectomized groups receiving increasing dose of 17β-estradiol decreased dose-dependently (Fig 3C). As shown in Fig 3D and 3E, the mRNA and protein expressions of Nav1.7 were significantly induced in the TMJ inflammation groups as compared with the control (*P* < 0.05), and Nav1.7 expression was further potentiated by 17β-estradiol dose-dependently (*P* < 0.05).

### Co-localization of ERα and ERβ with Nav1.7 in TG

**Fig 4:**
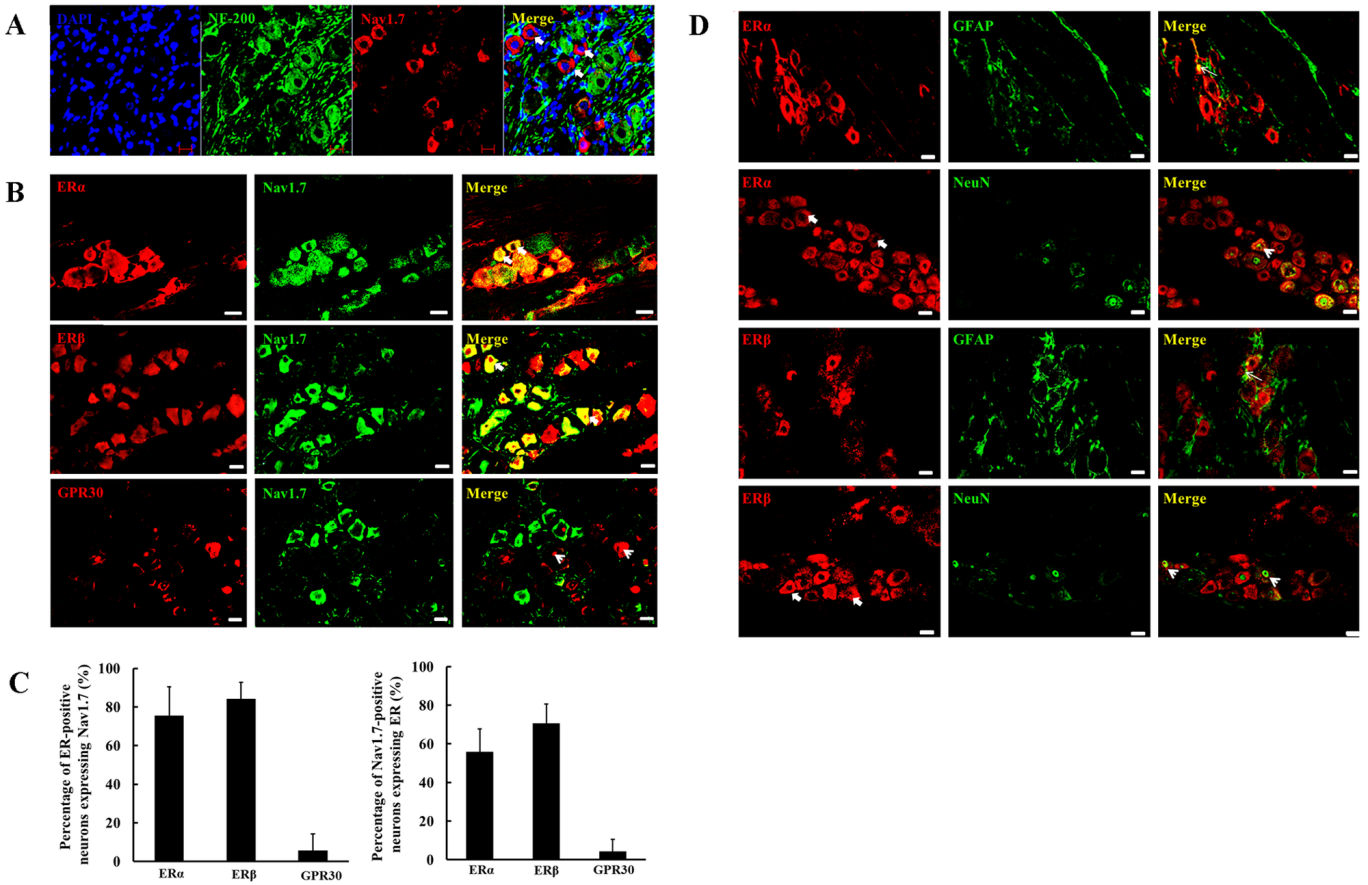
Co-localization of ERs and Nav1.7 in TG. (A) Representative immunofluorescence of Nav1.7 in TG. Nav1.7 was mainly located in the cytoplasm of small neurons, which were negative for NF200 (a marker for medium and large neurons), bars = 20 μm. (B) Representative immunofluorescence of Nav1.7 and ERα or ERβ or GPR30 in TG. Most of ERα or ERβ positive neurons exhibited co-localization with Nav1.7 (yellow, indicated by arrow); GPR30 positive neurons exhibited little co-localization with Nav1.7 (indicated by arrowhead), bars = 20 μm. (C) The percentage of Nav1.7 positive neurons expressing ERs and percentage of ERs positive neurons expressing Nav1.7. Data are expressed as mean ± SD. (D) Representative immunofluorescence of ERα or ERβ or GPR30 and NeuN or GFAP in TG. ERα or ERβ positive cells exhibited little co-localization with GFAP (labelled yellow, indicated by thin arrow) and a few of ERα or ERβ positive cells exhibited co-localization (labelled yellow, indicated by arrowhead) with NeuN (marker for neuronal nucleus), which indicated that ERα and ERβ were mainly located in cytoplasm of neurons (indicated by arrow), bars = 20 μm.

We first confirmed that Nav1.7 was mainly located in the small neurons, which showed negative staining of NF200 (medium and larger neuron marker) (Fig 4A), consistent with the previously study [33]. Moreover, 75.5% of ERα-positive neurons and 84.0% of ERβ-positive neurons expressed Nav1.7, whereas 55.9% and 70.6% of Nav1.7-positive neurons expressed ERα and ERβ, respectively, and almost no Nav1.7-positive neurons expressed GPR30 (Fig 4B and 4C). In Fig 4D, ERα and ERβ were mainly localized in the cytoplasm of neurons and some co-localized with NeuN (neuronal nucleus marker) in the nuclei of neurons, but very less co-localized with GFAP (glia marker) in the glia.

### Blocking estrogen receptors partially reversed hyperalgesia of inflamed TMJ and TMJ inflammation-induced upregulation of Nav1.7 in TG

**Fig 5:**
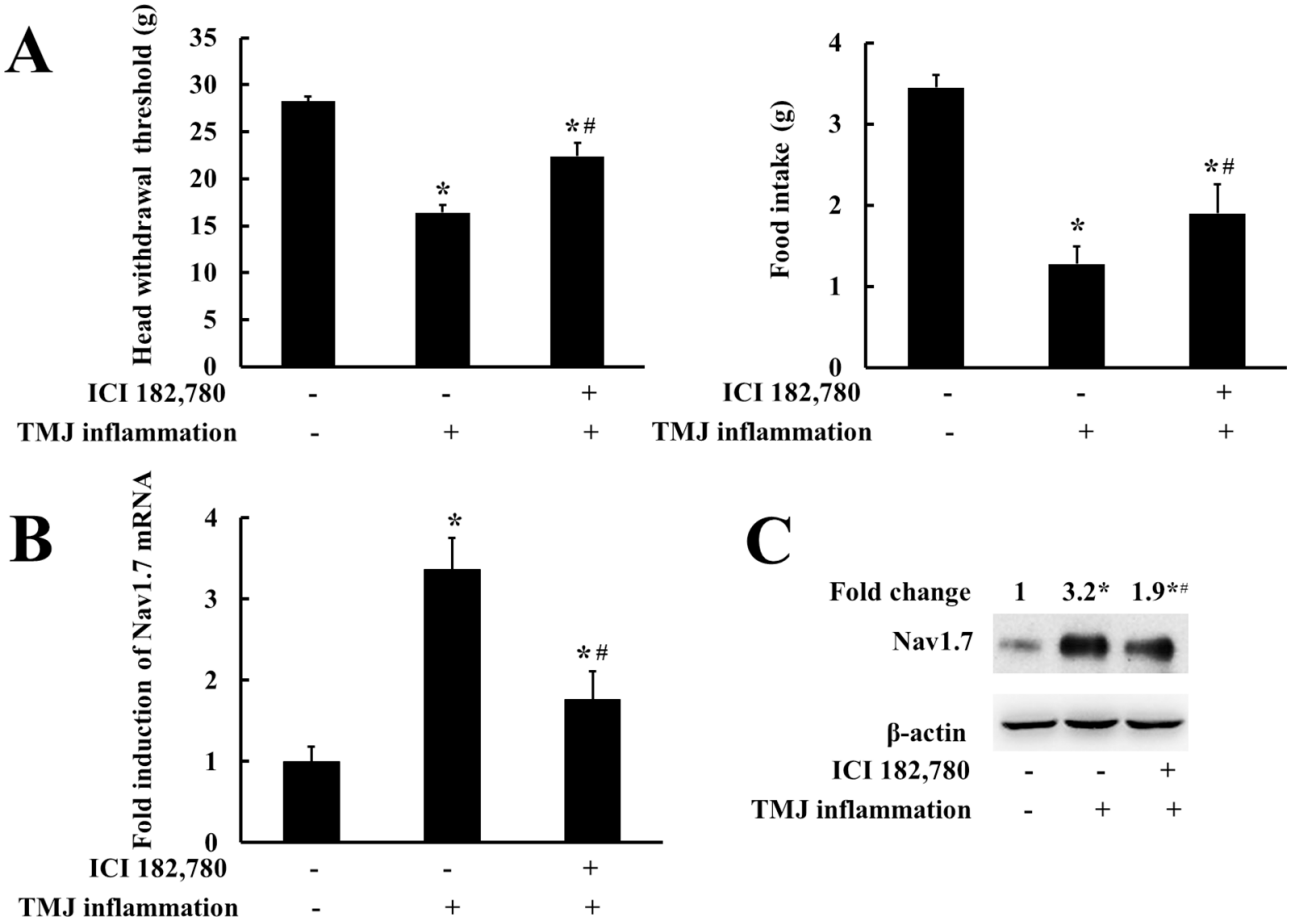
Blocking of estrogen receptors partially reversed hyperalgesia of inflamed TMJ and TMJ inflammation-induced expression of Nav1.7 in TG. (A) TMJ inflammation-induced in head withdrawal threshold and food-intake was partially reversed by blocking estrogen receptor. (B) TMJ inflammation-induced Nav1.7 mRNA expression was partially reversed after blocking estrogen receptor (n = 4, two-way ANOVA). (C) Representative immunoblotting for Nav1.7 expression in TG. TMJ inflammation-induced Nav1.7 protein expression was partially reversed by blocking of the estrogen receptor. β-Actin was served as an internal control for equal loading (n = 3). *P < 0.05 versus control group; ^#^*P* < 0.05 versus inflamed group. Data are expressed as mean ± SD.

We further examined whether ICI 182,780, antagonist for both ERα and ERβ, could reverse upregulation of Nav1.7 expression and hyperalgesia of inflamed TMJ in female rats. As shown in Fig 5A, the food intake and head withdrawal threshold were both decreased by TMJ inflammation, compared with the control group (*P* < 0.05). However, the decreases of the two parameters were partially reversed by pre-treatment of ICI 182,780 (*P* < 0.05). The induction of Nav1.7 mRNA and protein expression by TMJ inflammation were also partially reversed by ICI 182,780 in the TMJ inflammation group compared with that of the control group and the TMJ inflammation group without ICI 182,780 (Fig 5B and 5C).

### Estradiol enhanced Nav1.7 promoter activity

**Fig 6:**
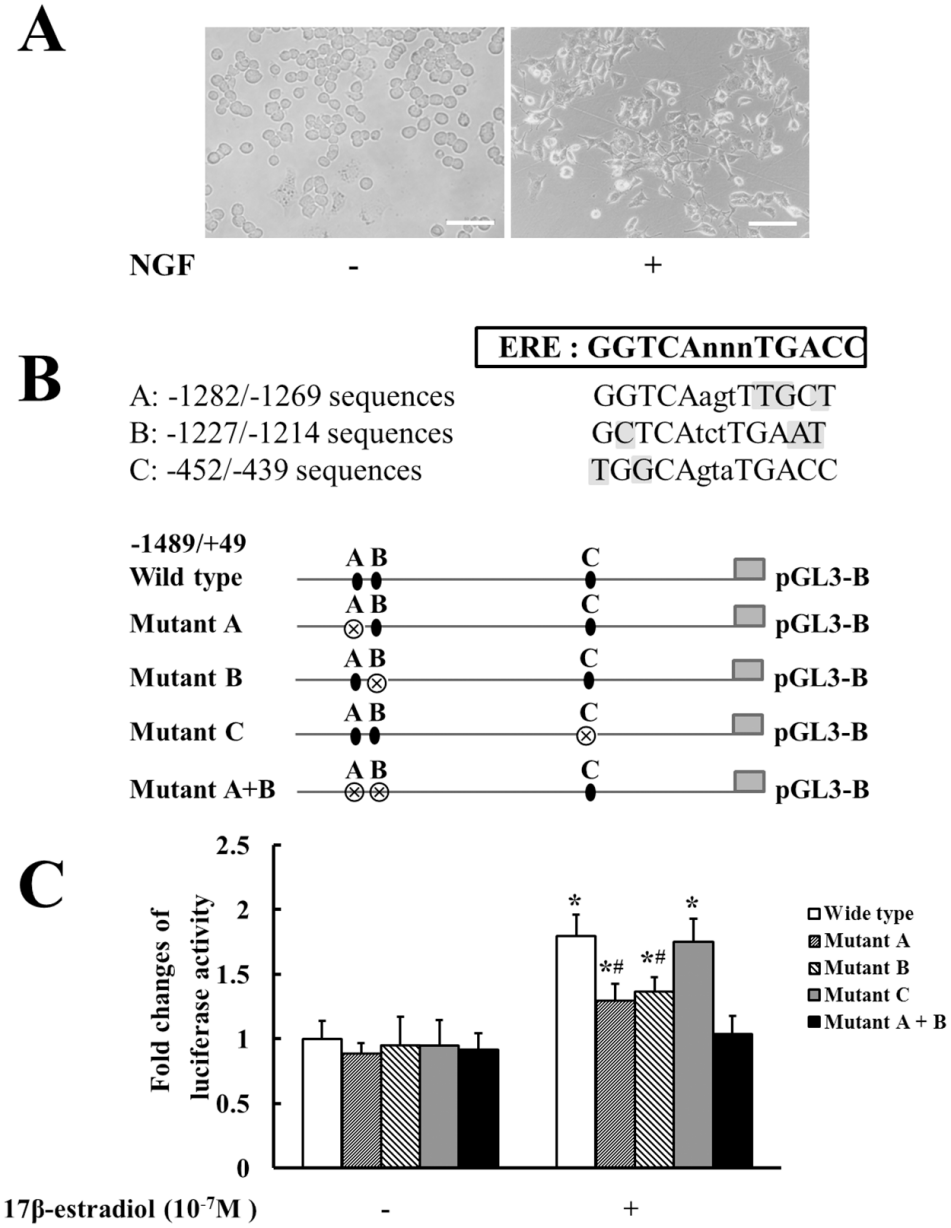
Estradiol enhanced Nav1.7 promoter activity. (A) Photomicrograph of representative PC12 cells differentiated with NGF for 24 h to resemble a neuronal phenotype. The NGF-induced PC12 cells extended obviously axon-like processes. (B) Diagram of the rat Nav1.7 promoter and corresponding mutants. The sequence of the ERE consensus is GGTCAnnnTGACC, three putative EREs in the promoter was correspondingly labelled as A, B, and C, and the shaded nucleotides was indicated as different from the ERE consensus. Black circle represents the putative ERE. Crossed circle represents mutated ERE. (C) 17β-estradiol effects on the activities of Nav1.7 wild-type promoter and mutants.17β-estradiol significantly enhanced the activities of wild-type promoter and the mutant with mutation of putative ERE at -452/-439, but completely failed to enhance the activity of the mutant with mutations of both putative EREs at -1282/-1269 and -1227/-1214. *P < 0.05 versus control group; ^#^*P* < 0.05 versus wild-type group. Data are expressed as mean ± SD.

The estrogen–ER complex binds to ERE in the promoter region of some gene, and then modulates the levels of associated mRNA and protein [21]. The essential ERE has the consensus sequence GGTCAnnnTGACC, a 13-nucleotide segment with 10 nucleotides forming an inverted repeat [7]. However, many genes have been found to contain sequences that appear to be ERE, most of these vary from the consensus by one or more nucleotides [34]. Three putative EREs were observed by sequence analysis in rat Nav1.7 promoter, including TGGCAgtaTGACC at -452/-439, GCTCAtctTGAAT at -1227/-1214, and GGTCAagtTTGCT at -1282/-1269. To examine whether estradiol regulated Nav1.7 expression through these EREs in the rat Nav1.7 promoter, reporter constructs containing the Nav1.7 promoter with the wild-type or mutant EREs were transfected into NGF-induced and ERα-transfected PC12 cells. As shown in Fig 6A, the NGF-induced PC12 cells extended obviously axon-like processes, which indicated that the PC12 cells have been differentiated to better resemble a neuronal phenotype. As shown in Fig 6B and 6C, 17β-estradiol significantly enhanced the activities of wild-type Nav1.7 promoter and the mutant with mutation of putative ERE at -452/-439, and less significantly enhanced the activities of mutants with mutation of ERE at -1282/-1269 alone or at -1227/-1214 alone, whereas 17β-estradiol completely failed to enhance the activity of the mutant with mutations of both putative EREs at -1282/-1269 and -1227/-1214.

## Discussion

In this study, we provided data for the first time to show that estradiol could modulate trigeminal ganglionic Nav1.7 expression to enhance hyperalgesia of inflamed TMJ. First, TMJ nociception and Nav1.7 expression in TG in female rats were correspondingly affected by physiological estradiol fluctuations during the estrous cycles. Second, estradiol dose-dependently upregulated Nav1.7 expression in TG and correspondingly enhanced TMJ nociception in the ovariectomized rats receiving increasing doses of estradiol. Third, estradiol further potentiated TMJ inflammation-induced Nav1.7 expression in TG and correspondingly enhanced hyperalgesia of inflamed TMJ. Fourth, the estrogen receptor antagonist, ICI 182,780, partially reversed inflammation-induced both hyperalgesia of inflamed TMJ and upregulation of Nav1.7 in TG. Fifth, estradiol enhanced the activity of Nav1.7 promoter depending on ERE at -1282/-1269 and -1227/-1214. These results suggested that estradiol could upregulate Nav1.7 expression in TG to contribute to hyperalgesia of inflamed TMJ.

Nav1.7 might play a role in sex difference of TMJ pain perception. Although the sex difference of TMD pain has been recognized for a long time and several mechanisms were proposed [3, 6, 35, 36], such difference remains to be fully understood. Here, we observed that trigeminal ganglionic Nav1.7 expression and the nociception of TMJ both co-ordinately fluctuated in the estrous cycle of female rats, in which the female rats especially showed the highest plasma level of estradiol and the lowest head withdrawal threshold in the proestrous phase. Furthermore, estradiol could upregulate Nav1.7 expression in TG in a dose-dependent manner in the ovariectomized rats. These results suggested that Nav1.7 as a critical pain related gene was a target for estradiol. These results also showed a positive relationship existed between Nav1.7 expression in TG and the sensitivity of TMJ to mechanical stimuli. Given that upregulation of Nav1.7 expression accompanies with its increased function for firing action potentials and amplifying weak stimuli in the neurons [12], it would be reasonable to believe that the estradiol-induced upregulation of Nav1.7 expression in TG could, to some extent, contribute to the increase in TMJ nociception. It is worth noting that the plasma levels of 17β-estradiol in the ovariectomized group treated with 0 μg of 17β-estradiol were not zero, but around 12.7 ± 2.7 pg/ml. The reason maybe that some organs and tissues such as fat, adrenal gland, brain tissue can also play some roles in the synthesis of estrogen to the blood circulation, but these estrogen don’t have the same effect of estrogen from ovarian secretion [37]. Considering that specific Nav1.7 blockers are under development to treat neuropathic pain [38], it is interestingly expected that these blockers would also treat TMD pain, and whether these blockers might be more effective in women than in men remains to be determined.

The effects of estradiol on Nav1.7 expression in TG and on TMJ nociception appeared to be more significant in the rats with TMJ inflammation than that in the rats without TMJ inflammation. During the estrous cycles, the fluctuations of head withdrawal threshold and Nav1.7 expression in TG were both modest, whereas the plasma levels of estradiol were more greatly fluctuated. Similarly, in the ovariectomized rats received different doses of 17β-estradiol, the change of head withdrawal threshold was well proportionally corresponding to that of Nav1.7 expression in TG, but less proportionally corresponding to that of plasma estradiol, under the condition without TMJ inflammation as shown in Fig 2. However, once there was TMJ inflammation as shown in Fig 3, the changes of nociceptive response and Nav1.7 expression in TG were much greater and more proportionally corresponding to that of plasma estradiol. These results suggested that estradiol combined with TMJ inflammation could more significantly affect Nav1.7 expression in TG and nociception of TMJ.

Estradiol potentiated TMJ inflammation-induced upregulation of trigeminal ganglionic Nav1.7 and hyperalgesia of inflamed TMJ through estrogen receptor signalling pathway. We observed in TG that 55.9% and 70.6% of the Nav1.7-positive neurons also expressed ERα or ERβ, respectively, whereas Nav1.7-positive neurons almost did not express GPR30. ERα and ERβ were confirmed to be expressed mostly in the neurons also expressing Nav1.7 in TG. These results provided a direct basis for estrogen to influence Nav1.7 expression more likely through the classical (genomic) mechanism of ER action. It was also consistently supported by our results of applying estrogen receptor antagonist ICI 182,780, which partially reversed TMJ inflammation-induced downregulation of mechanical threshold of the inflamed TMJ and upregulation of Nav1.7 expression. Our results of estrogen receptor antagonist are also consistent with a previous study, which shows that sex difference of inflammatory hypersensitivity is eliminated in mice lacking both the ERα and ERβ [39]. Based on these results, we suggested a possible new mechanism underlying estrogen involving in pain perception, i.e., estrogen receptor signalling pathway could affect Nav1.7 expression to contribute to hyperalgesia or sex difference of nociception. Certainly, in the future we need to identify whether ERα or ERβ or both were involved in estradiol-induced upregulation of Nav1.7 expression.

Based on the results that the Nav1.7-positive neurons in TG almost did not express GPR30, it appeared that the non-classical or non-nuclear mechanism of estrogen action might be hardly involved in upregulation of Nav1.7 expression in TG. However, it was not excluded that the non-classical mechanism of estrogen action might be involved in modulating Nav1.7 channel activity. For estrogen can trigger rapid activation of the MAPKs [9] and ERK1/2 can directly phosphorylate Nav1.7 and modulate its channel activity in neurons [40]. It will be an interesting research direction in the future to explore whether estrogen can affect Nav1.7 channel activity by the non-classical pathway.

Estradiol upregulated Nav1.7 expression through ERE in the promoter of Nav1.7. The human Nav1.7 promoter is known to be activated by NGF, phorbol esters, retinoic acid, and Brn-3a [41, 42]. However, it is little known about the regulation of rat Nav1.7 promoter. In the present study, we showed that estradiol could significantly enhance the activity of rat Nav1.7 promoter in NGF-induced and ERα-transfected PC12 cells. This result suggested that estradiol upregulated Nav1.7 mRNA and protein expressions in TG, at least in part, through its effect on Nav1.7 promoter. This result also supported that the upregulation of Nav1.7 expression by estradiol could be mainly depended on the classical (genomic) mechanism of ER action. Although three regions in the promoter of rat Nav1.7 appeared to be putative ERE according to sequence analysis, the putative ERE at -452/-439 was excluded, since mutations of both putative ERE at -1282/-1269 and -1227/-1214 in the promoter could completely reverse estradiol-induced enhancement of the Nav1.7 promoter activity, whereas mutation of the putative ERE at -452/-439 alone failed to reverse, and at -1282/-1269 alone or at -1227/-1214 alone only partially reversed estradiol-induced enhancement of the Nav1.7 promoter activities. These results suggested that the sequence of GGTCAagtTTGCT at -1282/-1269 and sequence of GCTCAtctTGAAT at -1227/-1214 in rat Nav1.7 promoter acted as ERE. Future study is needed to test whether estradiol could also activate human Nav1.7 promoter.

The Nav1.7 promoter is an important target for regulation of Nav1.7 expression or activity by other factors. Numerous cytokines, hormones and signalling cascades have been reported to regulate Nav1.7 mRNA levels [43]. For example, tumor necrosis factor-α [44], prostaglandin E2 [45], epidermal growth factor [46], brn-3a neuronal transcription factor [47] and so on were reported to upregulate Nav1.7 mRNA expression. Such effectors are thought to act primarily at the level of transcription or through its promoter. Certainly, some regulation may occur post-transcriptionally utilising mechanisms including mRNA splicing, editing and micoRNA expression. Therefore, the Nav1.7 promoter needs to be further explored.

Although we showed that the estradiol-induced upregulation of Nav1.7 mRNA and protein could be to some extend through enhancing Nav1.7 promoter activity, it is not ruled out that other mechanism might also involve in estradiol-upregulating Nav1.7 expression. For instance, estradiol might upregulate Nav1.7 expression through nerve growth factor (NGF), which can be upregulated by estradiol in our previous study [25], and is already known to be a positive regulator of Nav1.7 expression [41]. Estradiol might also upregulate Nav1.7 expression through NF-κB and cytokines, since NF-κB and its downstream cytokines can be potentiated by estradiol [5]. Those speculations might also some explain that the effects of estrogen and inflammation on Nav1.7 expression appeared synergistic rather than additive. The relationship between estrogen and Nav1.7 expression may be complicated and needs to be further explored.

In conclusion, our results showed that estradiol could potentiate trigeminal ganglionic Nav1.7 expression to enhance nociception of TMJ and hyperalgesia of inflamed TMJ. Our results may help better understand the sex-difference of TMD and even inspire the development of a new therapy for TMD pain.

## Supporting information

S1 File Individual data points. This is the individual data points underlying the graphs in each of the graphs presented.
(XLSX)
